# Effects of a recruitment maneuver on plasma soluble rage in patients with diffuse ARDS: a prospective randomized crossover study

**DOI:** 10.1186/cc14321

**Published:** 2015-03-16

**Authors:** M Jabaudon, N Hamroun, L Roszyk, R Blondonnet, R Guerin, JE Bazin, V Sapin, B Pereira, JM Constantin

**Affiliations:** 1 CHU Clermont-Ferrand, France

## Introduction

The soluble form of the receptor for advanced glycation endproducts (sRAGE) is a promising marker for epithelial dysfunction, but it has not been fully characterized as a biomarker during ARDS. Whether sRAGE could inform on the response to ventilator settings has been poorly investigated, and whether recruitment maneuver (RM) may influence plasma sRAGE remains unknown.

## Methods

Twenty-four patients with moderate/severe, nonfocal ARDS were enrolled in this prospective monocentric crossover study and randomized into a 'RM-SHAM' group when a 6-hour-long RM sequence preceded a 6-hour-long sham evaluation period, or a 'SHAM-RM' group (inverted sequences). Protective ventilation was applied, and RM consisted of the application of 40 cmH_2_O airway pressure for 40 seconds. Arterial blood was sampled for gas analyses and sRAGE measurements, 5 minutes pre RM (or 40-second-long sham period), 5 minutes, 30 minutes, 1 hour, 4 hours and 6 hours after the RM (or 40-second-long sham period).

## Results

**Figure 1:**
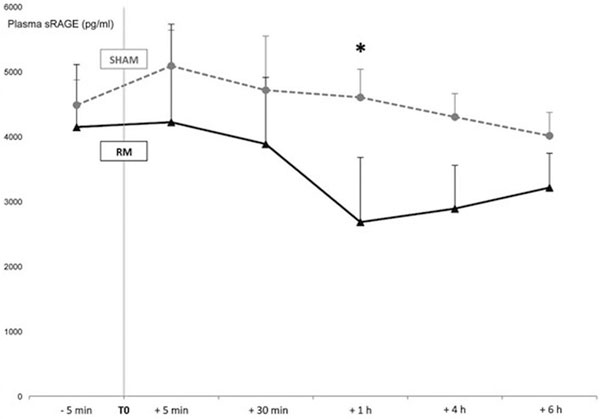
**Evolution of plasma levels of sRAGE (pg/ml) in both randomization sequences**.

Mean PaO_2_/FiO_2_, tidal volume, PEEP and plateau pressure were 125 mmHg, 6.8 ml/kg (ideal body weight), 13 and 26 cmH_2_O, respectively. Median baseline plasma sRAGE levels were 3,232 pg/ml. RM induced a significant decrease in sRAGE (-1,598 ± 859 pg/ml) in 1 hour (*P *= 0.043). At 4 and 6 hours post RM, sRAGE levels increased back toward baseline values. Pre-RM sRAGE was associated with RM-induced oxygenation improvement (AUC 0.87). See Figure 1.

## Conclusion

We report the first kinetics study of plasma sRAGE after RM in ARDS. Our findings could help to design future studies of sRAGE as a marker of response to therapeutic interventions during ARDS.

